# The Clinical Characteristics and Outcomes of Human Papillomavirus-Positive Nasopharyngeal Carcinoma in a Single-Institution Cohort

**DOI:** 10.3390/jcm12134264

**Published:** 2023-06-25

**Authors:** Muhammad Awawda, Saeed Salman, Salem Billan

**Affiliations:** Joseph Fishman Oncology Center, Rambam Health Care Campus, Haifa 3109601, Israel; m_awawda@rmc.gov.il (M.A.); sa_salman@rambam.health.gov.il (S.S.)

**Keywords:** nasopharyngeal carcinoma, head and neck neoplasms, human papillomavirus, p16, prognosis

## Abstract

Background: Nasopharyngeal carcinoma (NPC) is a head and neck cancer more frequent among East Asian populations compared with Western populations. While much is known about human papillomavirus’s (HPV’s) role in oropharyngeal cancer (OPC), little is known about its prevalence and prognostic value in NPC. The aim of this study is to investigate the role of HPV in NPC treated with definitive radiotherapy at a single institution. Methods: A retrospective cohort analysis of patient’s medical records and HPV status treated for NPC in Rambam Health Care Campus (Rambam HCC). Immunohistochemical staining for p16 was used as a surrogate marker of HPV infection in the tumor cells. All specimens were stained and evaluated by pathologists at the referring center independently. Results: In total, 87 patients diagnosed with NPC were treated at Rambam HCC between 2005 and 2018. Seventy-four patients had accessible data on the disease’s clinical parameters and p16 status. In total, 10/74 (13.5%) had p16-positive staining in tumor cells; 75% were men and over 50% were smokers. The average age of diagnosis for the whole cohort was 48 years, being lower for p16-positive patients compared with p16-negative patients at 43 and 49 years old, respectively. A total of 84% of the patients had advanced disease of stage III and IV at presentation. Only 16% were diagnosed with stage I and II. Unlike the p16-negative group, the p16-positive group did not include any stage I or II disease. In univariate and multivariate analysis of overall survival rates, the age at diagnosis and the nodal spread status were the only statistically significant measures. P16 status was not found to be associated with survival. Conclusions: The HPV prevalence in NPC is nontrivial. p16-positive patients had significantly less nodal spread and tended to be younger. Both age and nodal status were significantly correlated with the survival, but P16 status was not prognostic. Further large-scale trials are needed to elucidate the role of HPV in NPC.

## 1. Introduction

Nasopharyngeal carcinoma (NPC) is an epithelial carcinoma of the head and neck region, arising mostly within the posterolateral pharyngeal recess. It is twice as common in men than in women, with over 130,000 new cases yearly worldwide. In the past decade, the incidence and mortality have steadily declined, perhaps due to changes in etiological factors, as well as better and earlier diagnostic and therapeutic modalities [1,2].

Despite the histological similarity and anatomical proximity, it is distinctly different from other tumor subtypes originating from the head and neck mucosal lining, having different etiology, distribution, staging and treatment.

Among various etiological factors reported, viral oncogenes have been strongly implicated in the pathogenesis of NPC, being most frequently associated with Epstein–Barr virus (EBV), indicated by histopathological examination and geographical distribution. It is more common in EBV endemic regions such as East and Southeast Asia, making up over 70% of the newly diagnosed cases worldwide, of which 95% are of the non-keratinizing subtype that is known to be strongly associated with EBV [3,4].

Recently, multiple studies have supported the diagnostic and prognostic value of anti-EBV antibodies and free viral DNA in plasma and showed correlation to tumor burden. Ling-Long Tang et al. reported a non-inferior 3-year failure-free survival for patients with low-risk NPC, defined as stage II/T3N0M0 without adverse features, including Epstein–Barr virus DNA <4000 copies/mL treated with radiotherapy alone compared with standard chemoradiation [5]. Furthermore, radiotherapy dose de-escalation of 60 Gy guided by EBV DNA level to select low-risk NPC patients after induction chemotherapy (IC) showed favorable survival outcomes and a limited toxicity profile [6].

Almost all NPC cells demonstrate monoclonal EBV episomes in tumor cells. The proposed pathomechanism of carcinogenesis is through the expression of type II EBV latency gene products (e.g., LMP1, LMP2, EBNA1) facilitating global hyper-methylation, altering multiple cellular pathways and host microenvironments, thus promoting clonal expansion, proliferation and oncogenesis [7].

Human papilloma virus (HPV) is a well-known causative factor for oropharyngeal carcinoma (OPC). Nowadays, it is found in over 70% and 50% of the newly diagnosed cases in the United States and the United Kingdom, respectively [8]. Recently, it has been proposed as a causative agent in a minority of NPC cases, mainly occurring among white people of European descendant [9,10,11].

HPV is a small 55 nm diameter non-enveloped, circular double-stranded deoxyribonucleic acid (DNA) oncogenic virus, best known to be a sexually transmitted disease (STD) having a strong tropism for host mucosal and cutaneous stratified squamous epithelium. It has been shown to cause genital warts, pre-cancerous lesions and cancers among different body sub-sites, mainly gynecological malignancies (cervical, vaginal and vulvar cancer), anal cancer and head and neck cancers; it mostly affects sites harboring lymphoid tissue of the Waldeyer’s ring (Adenoids, Tubal, Palatine and Lingual tonsils), occurring predominantly within the Oropharynx [12].

Multiple HPV strains have been identified. They are divided into low-risk non-cancerous strains (e.g., 6, 11) causing indolent lesions, and high-risk aggressive strains causing cancers; 14 of these strains are the most prevalent (16, 18, 31, 33, 35, 39, 45, 51, 52, 56, 58, 59, 66 and 68), where 16 and 18 are responsible for over 70% of HPV-related cancers [13].

HPV exerts its oncogenic effect mainly through the integration of the HPV genome into the host chromosome, subsequently allowing for the expression of the viral E6 and E7 genes so that their oncoproteins disrupt the tumor suppressor genes P53 and RB, respectively, resulting in dysregulated and selective growth advantage [12].

Although HPV-positive oropharyngeal carcinoma (OPC) is strongly known to behave differently from HPV-negative OPC, the clinical behavior and prognostic value of HPV in NPC has been controversial, as trials have shown contradictory results [9,14,15,16,17,18].

Traditionally, the treatment of locally advanced NPC has been definitive radiotherapy or chemoradiotherapy with neo-adjuvant or adjuvant chemotherapy depending on the disease stage at presentation, regardless of HPV and EBV status, even though the latter has been shown to be prognostic [19,20,21,22]. Despite multimodal treatment, 10–15% and 20–30% of patients will suffer from loco-regional recurrence (LRR) and distant metastasis, respectively [23,24].

This treatment approach has largely been shaped by large-scale trials from endemic regions. Nevertheless, it has been adopted by most centers even in non-endemic areas, mainly due to the lack of deep understanding of the differences in the disease behavior and response to treatment between endemic and non-endemic NPC. For instance, despite two previous pivotal trials [20,21] in endemic countries showing favorable outcomes with the addition of induction chemotherapy prior to concurrent chemoradiotherapy, Ou et al. showed that induction chemotherapy with docetaxel, cisplatin and fluorouracil did not improve overall survival or progression-free survival for non-endemic NPC [25].

Additionally, the histological subtypes’ distribution differs, as non-endemic countries have a higher prevalence of keratinizing squamous cells carcinoma compared with non-endemic countries where the undifferentiated subtype with a better prognosis predominates. As we are eager to allow precision medicine for all, there is an ongoing need to deepen our understanding of the various etiological and biological factors associated with the disease.

The aim of this study is to retrospectively investigate the prevalence, clinical outcomes and prognostic value of HPV among patients diagnosed with non-metastatic NPC treated with definitive radiotherapy or chemoradiotherapy at our institution.

## 2. Materials and Methods

### 2.1. Study Population

Our study received an institutional research ethics board approval. All patients who were newly diagnosed with nasopharyngeal carcinoma and treated with curative intent using external beam radiotherapy between the years 2005 and 2018 in Rambm Health Care Camps (the tertiary cancer center of north Israel) were included. All patients had clinical and imaging follow-up at our institution as stated by the NCCN guidelines dated for the same period. Patients with distant metastasis were excluded from the study as well as patients for whom tissue samples were unavailable for examination of the viral status.

### 2.2. Histopathological Analysis

The viral etiology was confirmed using p16 immunohistochemistry staining (≥70% strong nuclear and cytoplasmic staining), a surrogate marker of prior HPV infection in the tumor tissue. The tissue samples’ evaluations were made by head and neck pathologists from 10 different institutes across Israel, each made by pathologists at the local institutes of the initial tissue sampling.

### 2.3. Treatment and Outcome Assessment

Patients’ clinical characteristics, demographics, treatments and outcome data were retrieved from the local electrical medical records database. All tumors were staged using the AJCC TNM Classification eighth edition, which were based on the initial radiology reports at diagnosis. A multidisciplinary team comprising radiation oncologists, medical oncologists and an otorhinolaryngology surgeon treated our patients. Fifteen patients were treated with 3D conformal radiotherapy and the rest of the patients were treated with intensity-modulated radiotherapy (IMRT) with or without chemotherapy. Patients who had locally advanced diseased of T category 3 or 4 or having lymph node–positive NPC received concurrent chemoradiotherapy with additional induction chemotherapy. Patients with T1N0 were treated with radiotherapy alone and patients with T2N0 were managed with concurrent chemoradiotherapy without additional induction chemotherapy.

The tumor volume receiving a radiation dose of 70 Gy and the electively treated volume irradiated to 56 Gy were contoured by senior head and neck radiation oncologist based on the Radiation Therapy Oncology Group (RTOG) contouring guidelines. The delineation and treatment plans were reviewed in weekly quality-assurance staff meetings.

The treatment response was assessed initially 4 weeks after radiotherapy by radiation oncologist and otorhinolaryngology surgeon. Patients were followed every 3 months for two years, every 4 months for the third year and every 6 months for 2 more years, and annually thereafter. Disease recurrences were diagnosed by radiologic findings and confirmed histologically whenever the findings were unequivocal.

The patients’ demographics and clinical characteristics, as well as disease-free survival (DFS), disease-specific survival (DSS) and overall survival (OS) were compared for the HPV-positive and HPV-negative patients.

The follow-up time was assessed from the date of diagnosis until November 2019.

### 2.4. Statistical Methods

Five-year overall survival (OS), disease-specific survival (DSS) and disease-free survival (DFS) were calculated using the Kaplan–Meier method, and differences in survival rates were assessed using the log-rank test. OS was measured from the date of surgery to the date of death or the last follow-up. For DSS, patients who died from causes other than NPC were censored at the time of death. All variables that had prognostic potential were subjected to both univariate and multivariate analyses using the Cox proportional hazards regression model. Analyses were performed using IBM SPSS Statistics for Windows, version 24 (IBM Corp., Armonk, NY, USA). All statistics were two-sided. A value of *p* < 0.05 was considered to indicate statistical significance. Differences between p16-positive and p16-negative cohorts were evaluated by applying Fisher’s exact test for categorical variables.

## 3. Results

**Figure 1:**
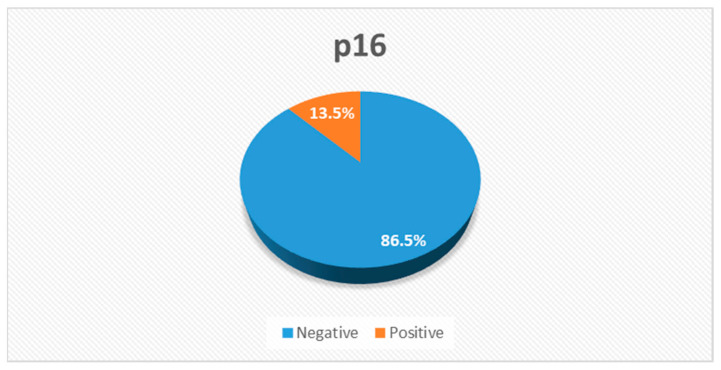
IHC p16 prevalence.

**Figure 2:**
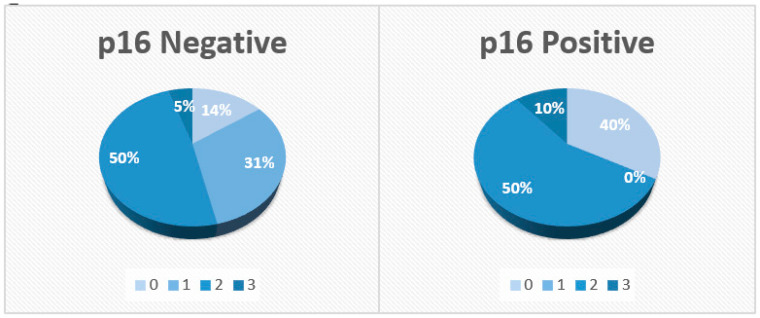
Nodal involvement stratified by p16 status.

**Figure 3:**
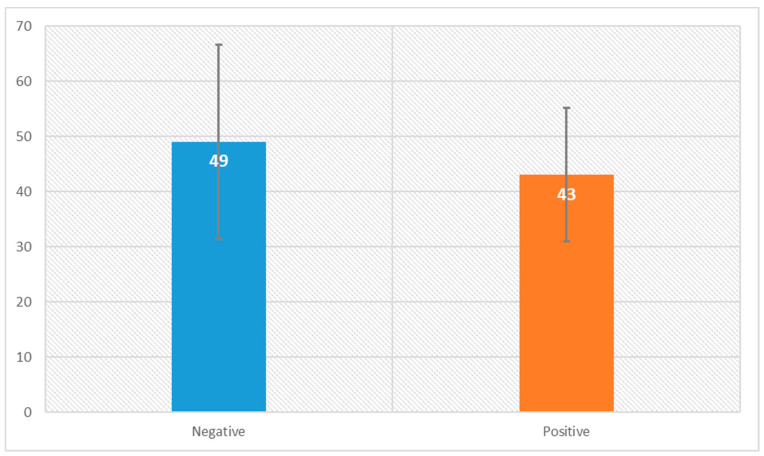
Age distribution stratified by p16 status.

A total of 87 patients were diagnosed with NPCs and treated with radiotherapy between the years 2005 and 2018. Of these, there were 74 metastasis-free patients for whom we had access to their immunohistochemistry (IHC) slides for p16 stain, which were positive for 10 (13.5%) of these individuals (Figure 1). The median follow-up time from the date of diagnosis was 78 months. Of the 74 patients, 78.4% were male patients and 57% were non-smokers. A total of 92% received induction chemotherapy, consisting mostly of two cycles of three weekly cisplatin and fluorouracil chemotherapy treatments. Concurrent chemotherapy, consisting mainly of weekly cisplatin, was administered to 97.2% of the patients, while only two patients (2.8%) with disease stage T1N0 received radiation therapy alone. During the follow-up period, 14 patients (18.9%) had disease recurrence, of whom 11 (78.6%) died. Table 1 shows the clinical and pathological characteristics of the patients. Of the 64 patients with P16-negative disease, 76.5% were male patients and 36% were smokers. A total of 17% had T1 category disease, 25% had T2, 34.4% had T3 and 23.4% had T4. While 86% had node-positive disease, 31% had N1 category, 50% had N2 and 5% had N3. Of the 10 patients with p16-positive disease, 90% were male patients and 60% were smokers. A total of 40% had T4 category with T1, T2 and T3 comprising 20% each, while 60% had node-positive disease as follows, 50% N2 and 10% N3. When comparing p16-positive to p16-negative patients, we found that T category (*p* value 0.721), smoking status (*p* value 0.298) and female-to-male ratio (*p* value 0.31) were similar. On the contrary, patients who were p16-positive had statistically significantly (*p* value 0.036) less nodal spread compared with those who were p16-negative (Figure 2). Although not statistically significant, p16-positive patients tended to be younger, with a mean age at diagnosis of 49 years and 43 years for the P16-negative and p16-positive patients, respectively (Figure 3).

**Figure 4:**
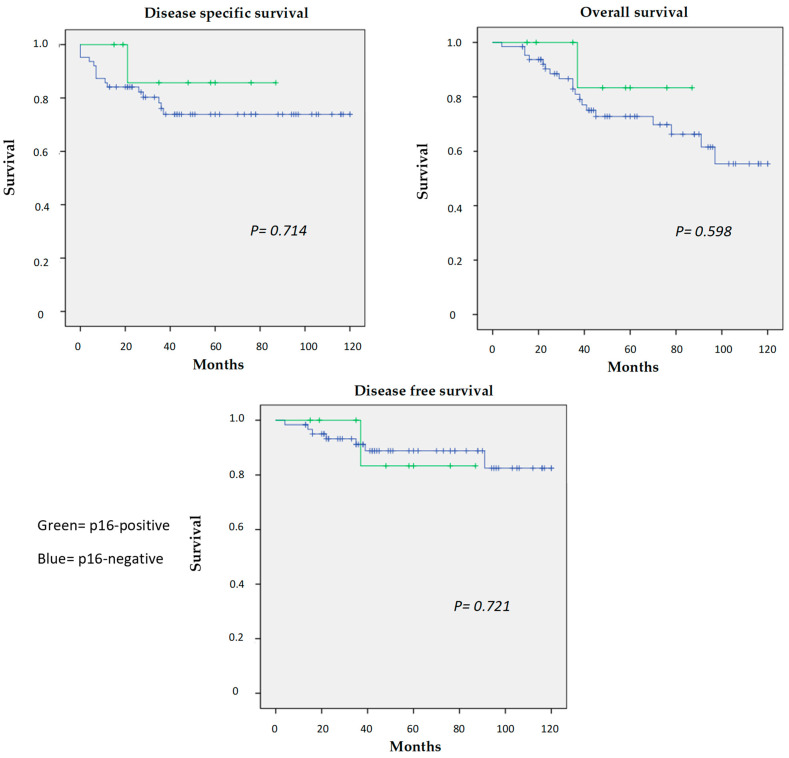
Kaplan–Meier survival estimates.

The oncological outcomes for all patients are illustrated in Figure 4. The overall 5-year survival for the entire cohort was 73.5%. At 5Y, the OS, DSS and DFS for p16-negative patients were 73.3%, 81.8% and 79.1%, respectively, as compared with 87.5%, 87.5% and 78.8% for those who were p16-positive (non-statistically significant). In univariate analyses (UVA), the patient age (*p* value 0.002) and the N category (*p* value 0.034) were predictors for overall survival, older age was also a predictor for disease-specific mortality (*p* value 0.034) and advanced T category was associated with disease recurrence (*p* value 0.032).

On multivariate analyses (MVA), the patient age (*p* value 0.017) and the N category (*p* value 0.028) were also predictors for overall survival, but older age was not a predictor for disease-specific mortality anymore (*p* value 0.089). Advanced T category was still associated with disease recurrence, having a roughly five-fold relative risk (*p* value 0.043). There was an over 3-fold increase in mortality for patients over 60 compared with those who were younger. The p16 status was not associated with OS, DSS or DFS either on univariate analyses or on multivariate analyses (Table 2).

## 4. Discussion

Our study investigated the prevalence and prognostic value of HPV in seventy-four non-metastatic NPC patients, of whom over 90% were treated with induction chemotherapy followed by definitive chemoradiotherapy.

In total, 20% had disease recurrence during the follow-up period, of whom 78% had cancer-related death. Using p16 as a surrogate marker for HPV in NPC, we found that 13.5% (10/74) of our patients’ biopsy specimens stained positive for p16.

HPV is strongly associated with head and neck cancer, particularly with OPC and, to a lesser extent, with oral cavity cancer and NPC. Though its significance is still debatable, its prevalence among NPCs varies widely from below 10% to over 50%, with white people and Caucasian people showing the most prominent associations [9,10,11].

There are approximately 14 high-risk HPV strains. While HPV-16 is the predominant type in OPC, occurring in over 95% of these cases, over 60% of HPV-positive NPC patients harbor other high-risk HPV types [11].

Several detection methods for HPV exist. Although in situ hybridization (ISH) and polymerase chain reaction (PCR) are the most sensitive techniques, they are complicated, expensive and lack specificity for causal etiology [9]. On the contrary, p16 detection using immunohistochemistry (IHC) is a strong surrogate marker for HPV in OPC and is still commonly practiced due to its wider availability and lower cost [15]. Walline et al. have suggested that p16 could serve as a good surrogate marker for HPV in NPC as well. Using ISH and PCR, they showed that eight out of nine p16-positive patients were also HPV-positive [11].

Moreover, p16 positivity might indicate that the virus is transcriptionally active, suggesting that the virus is a driving factor rather than being merely a bystander infection. This causal relationship assumption is further enhanced by the rare existence of EBV and HPV co-infection in the same tumor cells [15,26] and the high correlation between the viral status and the NPC histological WHO type.

While EBV is recognized as the primary etiologic factor in non-keratinizing NPC WHO type II and III, WHO type I keratinizing NPC lacks an association with EBV and is more linked to other etiological factors such as smoking and HPV. In a study of non-endemic NPC, all of the EBV-positive tumors were non-keratinizing type II and III NPC, while only two-thirds of the HPV+ NPC were as such, and two-thirds of the EBV/HPV-negative tumors were keratinizing type I NPC [15]. Nearly 95% of our cohort had WHO type II and III NPC.

The clinical presentation of HPV+ NPC seems to be different than HPV-negative NPC. Windon et al. have shown that in comparison to HPV + OPC and HPV − NPC, the HPV + non-OPC has uniqueness, a different presentation and perhaps a worse prognosis [14]. Huang et al. reported a predilection for a central location of the HPV-positive tumor within the nasopharynx, a trend toward a higher involvement of level IB nodes and a higher pain score [27]. Verma et al. found that viral etiology did not affect outcomes among 352 NPC patients, of whom less than 10% had HPV-positive disease, but HPV + NPC population was enriched with advanced T category and smokers [28]. In our study, p16-positive patients were enriched with smokers as well (60%), compared with only 37% in the p16-negative subgroup, though statistical significance was not reached. The T category was not different between the subgroups, but p16-positive NPC had less nodal spread and tended to be younger, in accordance with previous reports on NPC [10,29] but in contrast with HPV + OPC, which usually presents with a smaller primary tumor and a prominent nodal spread. Despite having less nodal spread, all of our patients with p16 stains presented in a locally advanced stage (III or IV).

In contrast to its favorable prognostic value in OPC, HPV positivity remains controversial in non-OPC because even though several studies have suggested HPV to have a favorable impact on the prognosis of head and neck cancer despite sub-site, others have shown no survival benefit or even a detrimental effect among HPV-positive non-OPC patients [9,14,15,16,17,18]. We did not find any prognostic implication for p16, but age and N category were predictors for overall survival and the T category was predictive for disease recurrence, similarly to previous reports [30,31]. Interestingly, Wotman et al. have indicated an association between HPV positivity and race-dependent NPC prognosis, being more favorable among East Asian people and worse among Caucasian people [10]. Others suggested p16 to have an independent prognostic impact on NPC regardless of HPV status [32]. Stephen et al. reported that p16-positive NPC who were HPV-positive as well tended to have better survival than p16-positive in an HPV-negative NPC [33].

Unlike EBV + NPC, in which the predominant pattern of failure is distant metastasis, Stenmark et al. reported higher loco-regional recurrence rates among HPV + NPC [15]. This observation has to be further investigated, as it raises several imperative questions, such as whether there is a need for intensifying the local treatment applied with escalating the definitive chemoradiotherapy or if HPV + NPC should receive a less favorable AJCC staging compared with EBV + NPC with the same clinical characteristics. This need is contradictory to the trend in HPV-positive OPC, where the disease gets a more favorable staging, is more radiosensitive and many clinicians recently have opted for treatment de-escalation. The decision is of crucial importance, as the currently applied treatment is highly toxic with a decreased long-term quality of life.

Furthermore, we found patients older than 60 years to have a three-fold relative risk of mortality. Nearly 80% of NPC cases occur in individuals aged 30–60 years. At our institution, 30% of NPC cases are in patients older than 60 years. A small amount of data has been published regarding the appropriate definition of elderly in NPC. Additionally, it is arguable whether the chronological age or the biological age has to be considered. Previous retrospective and prospective trials have chosen various age cutoffs where 60 was the lowest limit and a commonly used definition [34,35]. Studies have shown elderly patients over 60 years to have worse survival than younger patients and that it becomes worse as age increases [36]. On the other hand, when adjusting for confounding factors such as disease stage, smoking, comorbidities and treatment intensity, no significant differences in terms of survival were observed. Hence, age has to be considered in the setting of the specific host and tumor-related features rather than independently [37]. Moreover, elderly patients tend to respond differently to the treatment; Wang et al. showed that the addition of induction chemotherapy had no impact on survival compared to chemoradiotherapy alone and led to worse grade III to IV toxicities [38]. In the MAC-NPC meta-analysis, elderly patients (≥60 years) constituted only 13% of the total cohort and chemotherapy had a detrimental effect on the OS of elderly NPC patients [39].

The incidence of the HPV-related malignancies, particularly gynecological cancer, has declined dramatically after the introduction of the HPV vaccine, which is recommended at a young age to confer an effective prevention method against the sexually transmitted virus. Diana et al. found the vaccination to significantly lower the prevalence of oral HPV infection, with an estimated efficacy of 90%, but low vaccine coverage remains a major issue [40]. Yet, there was not a parallel decline in NPC incidence, which needs to be further investigated.

Recently, several novel vaccine-based therapies and cellular therapies have shown promise for the treatment of HPV-related head and neck malignancies. Some are being tested in combination with immune-checkpoint inhibitors, especially PD-1 inhibitors such as the biologic CUE-101 causing selective expansion of the HPV16 E711-20-specific population of cytotoxic CD8 + T cells [41]. Thus, the awareness of the role of HPV in NPC is of huge importance, as those patients may be candidates for a targeted therapy in the future or be enrolled in clinical trials.

Additionally, the association between HPV and NPC raises concerns whether or not the nasopharynx has to be irradiated electively in HPV+ head and neck cancer of unknown primary. We believe this might be highly relevant for patients whose risk of having HPV+ NPC is non-negligible, particularly among non-endemic populations, Caucasian people, smokers and patients with nodal involvement patterns consistent with NPC, such as level V [15].

Further large-scale studies are needed to validate whether p16 can serve as a good surrogate marker for HPV infection in NPC, or if advanced methods, such as PCR or ISH, should be performed. Equally, NPC should perhaps be categorized into viral etiological subgroups. The nature, clinical differences and molecular profiles of the EBV + HPV−, EBV − HPV−, EBV − HPV+ and the rarely-occurring EBV + HPV + NPC types should be investigated.

In conclusion, HPV-positive NPC is assumed to be a distinct clinical entity different from other NPC or HPV-positive OPC. Its prognostic implication has to be studied while considering different confounding factors possibly affected by HPV status such as age, smoking status, histological type and disease stage.

## 5. Strengths

All of our patients were treated at a single medical facility, mostly receiving induction chemotherapy followed by chemoradiotherapy, and then followed for a relatively long follow-up period at our institution.

## 6. Limitations

Due to the nature of our study, there could be a bias, which applies to all retrospective studies.

Additionally, our study lacked EBV status, HPV subtyping, standardized detection methods and centralized labs.

## Figures and Tables

**Table 1:** Baseline characteristics of p16-positive and -negative patients.

Patients		p16-Negative	p16-Positive	*p* Value
**Number**		64	10	
**Age ± SD (years)**		49 ± 17.65	43 ± 12.1	0.68 ^a^
**F:M**		15:49 (23%)	1:9 (10%)	0.31 ^a^
**T category**				0.721 ^a^
	**1**	11	2	
	**2**	16	2	
	**3**	22	2	
	**4**	14	4	
**N category**				**0.036** ^a^
	**0**	9	4	
	**1**	20	0	
	**2**	32	5	
	**3**	3	1	
**N status (positive)**		55/64 (86%)	6/10 (60%)	0.067 ^a^
**Smoking**		23/61 (37%)	6/10 (60%)	0.298 ^a^

^a^—Fisher’s exact test.

**Table 2:** Univariate (UVA) and multivariate (MVA) survival analyses.

	Overall Survival			
	UVA		MVA	
Variable	RR	*p*	RR	*p*
Age > 60	3.441 (1.555–7.613)	0.002	3.045 (1.216–7.624)	0.017
T late vs. early	1.481 (0.635–3.476)	0.361	1.384 (0.557–3.439)	0.484
N category	ref	0.034	ref	0.028
1 vs. 0	1.24 (0.425–3.624)	0.694	0.983 (0.319–3.035)	0.977
2 vs. 0	0.335 (0.102–1.107)	0.073	0.389 (0.114–1.323)	0.13
3 vs. 0	1.909 (0.456–8)	0.376	3.483 (0.746–15.276)	0.113
p16	0.604 (0.142–2.57)	0.464	0.644 (0.125–3.309)	0.598
	**Disease-specific survival**			
	**UVA**		**MVA**	
**Variable**	**RR**	***p***	**RR**	***p***
Age > 60	3.273 (1.097–9.769)	0.034	2.930 (0.85–10.101)	0.089
T late vs. early	2.528 (0.695–9.188)	0.159	2.297 (0.623–8.462)	0.212
N category	ref	0.741	ref	0.699
1 vs. 0	0.823 (0.183–3.707)	0.799	0.687 (0.145–3.258)	0.636
2 vs. 0	0.493 (0.117–2.074)	0.333	0.592 (0.134–2.622)	0.49
3 vs. 0	1.054 (0.11–10.140)	0.964	2.019 (0.19–21.431)	0.56
p16	0.558 (0.072–4.296)	0.575	0.667 (0.077–5.812)	0.714
	**Disease free survival**			
	**UVA**		**MVA**	
**Variable**	**RR**	***p***	**RR**	***p***
Age > 60	2.806 (0.973–8.091)	0.056	2.726 (0.809–9.188)	0.106
T late vs. early	5.155 (1.154–23.039)	0.032	4.755 (1.052–21.504)	0.043
N category	ref	0.592	ref	0.819
1 vs. 0	0.679 (0.17–2.716)	0.584	0.85 (0.194–3.723)	0.829
2 vs. 0	0.401 (0.108–1.495)	0.173	0.601 (0.151–2.391)	0.47
3 vs. 0	0.699 (0.078–6.266)	0.749	1.41 (0.139–14.331)	0.771
p16	1.056 (0.236–4.72)	0.943	1.363 (0.25–7.442)	0.721

## Data Availability

Data are available upon request from the authors.
